# Trabecular architecture in the sciuromorph femoral head: allometry and functional adaptation

**DOI:** 10.1186/s40851-018-0093-z

**Published:** 2018-05-15

**Authors:** Maja Mielke, Jan Wölfer, Patrick Arnold, Anneke H. van Heteren, Eli Amson, John A. Nyakatura

**Affiliations:** 1 AG Morphologie und Formengeschichte, Institut für Biologie und Bild Wissen Gestaltung. Ein interdisziplinäres Labor, Humboldt-Universität zu Berlin, Unter den Linden 6, Berlin, 10099 Germany; 2 Institut für Zoologie und Evolutionsforschung mit Phyletischem Museum, Ernst-Haeckel-Haus und Biologiedidaktik, Friedrich-Schiller-Universität Jena, Erbert-Straße 1, Jena, 07743 Germany; 3 Department of Human EvolutionMax Planck Institute for Evolutionary Anthropology, Deutscher Platz 6, Leipzig, 04103 Germany; 4 Sektion Mammalogie, Zoologische Staatssammlung München – Staatliche Naturkundliche Sammlungen Bayerns, Münchhausenstr. 21, München, 81247 Germany; 5 GeoBio-Center, Ludwig-Maximilians-Universität München, Richard-Wagner-Str. 10, München, 80333 Germany; 6 Department Biologie II, Ludwig-Maximilians-Universität München, Großhaderner Str. 2, Planegg-Martinsried, 82152 Germany

**Keywords:** Allometry, Functional adaptation, Lifestyle, Sciuromorpha, Trabecular bone, 3D microstructure

## Abstract

**Background:**

Sciuromorpha (squirrels and close relatives) are diverse in terms of body size and locomotor behavior. Individual species are specialized to perform climbing, gliding or digging behavior, the latter being the result of multiple independent evolutionary acquisitions. Each lifestyle involves characteristic loading patterns acting on the bones of sciuromorphs. Trabecular bone, as part of the bone inner structure, adapts to such loading patterns. This network of thin bony struts is subject to bone modeling, and therefore reflects habitual loading throughout lifetime. The present study investigates the effect of body size and lifestyle on trabecular structure in Sciuromorpha.

**Methods:**

Based upon high-resolution computed tomography scans, the femoral head 3D inner microstructure of 69 sciuromorph species was analyzed. Species were assigned to one of the following lifestyle categories: arboreal, aerial, fossorial and semifossorial. A cubic volume of interest was selected in the center of each femoral head and analyzed by extraction of various parameters that characterize trabecular architecture (degree of anisotropy, bone volume fraction, connectivity density, trabecular thickness, trabecular separation, bone surface density and main trabecular orientation). Our analysis included evaluation of the allometric signals and lifestyle-related adaptation in the trabecular parameters.

**Results:**

We show that bone surface density, bone volume fraction, and connectivity density are subject to positive allometry, and degree of anisotropy, trabecular thickness, and trabecular separation to negative allometry. The parameters connectivity density, bone surface density, trabecular thickness, and trabecular separation show functional signals which are related to locomotor behavior. Aerial species are distinguished from fossorial ones by a higher trabecular thickness, lower connectivity density and lower bone surface density. Arboreal species are distinguished from semifossorial ones by a higher trabecular separation.

**Conclusion:**

This study on sciuromorph trabeculae supplements the few non-primate studies on lifestyle-related functional adaptation of trabecular bone. We show that the architecture of the femoral head trabeculae in Sciuromorpha correlates with body mass and locomotor habits. Our findings provide a new basis for experimental research focused on functional significance of bone inner microstructure.

**Electronic supplementary material:**

The online version of this article (10.1186/s40851-018-0093-z) contains supplementary material, which is available to authorized users.

## Background

Sciuromorpha represent an expedient group for studying morphological adaptations to functional constraints, as they display diverse locomotor habits. They comprise more than 300 species with a wide range in body size [1] and which have adopted different locomotor behaviors (termed ’lifestyles’ in this study), which can be classified into arboreal, fossorial, aerial, and semifossorial. Whereas arboreal species live and nest in trees and spend little time on the ground, fossorial ones are adapted to a ground-dwelling lifestyle, digging burrows and nesting in the ground [2]. The fossorial lifestyle most probably evolved multiple times independently in the sciuromorph clade [2, 3]. Aerial (gliding) squirrels acquired a patagium enabling the animals to glide over long distances [4]. Members of the genus *Tamias* (chipmunks) adopted an intermediate lifestyle between arboreal and fossorial. They dig subterranean burrows for nesting, but climb trees when escaping predators [5]. They are thus called ’semifossorial’ in this study.

These lifestyle categories are expected to differ in directional variability, frequency and amount of loading. Arboreal habitats are diverse with respect to substrate slope, branch- or trunk diameter and texture. They require both climbing and jumping abilities, and impose diverse loading patterns on the limb bones (e.g. [6]). Aerial sciuromorphs, being mostly arboreal themselves, are found in habitats of similar diversity. However, gliding requires increased bone lightness and endurance in spanning the patagium. Additionally, they need to sporadically generate particularly large take-off forces and withstand particularly large landing forces (e.g. [7]). Fossorial species are expected to experience a more uniform loading than arboreal ones, as terrestrial locomotion and digging behavior are most demanding in developing high speeds and large unidirectional forces, respectively (e.g. [6]).

Different locomotor habits demand appropriate morphological adaptations to the characteristic loading patterns. Trabecular, or cancellous, bone enables such adaptations to loads acting on the skeleton of vertebrates [8–13]. The thin bony struts, called trabeculae, build an anisotropic microstructure that offers both stability and a light-weight construction [14]. The principal trabecular orientation often corresponds to the main stress trajectories within the bone (e.g. [15]) and therefore provides resistance against loads acting on it. The initial structure of trabecular bone in individuals is genetically regulated, and thus inherited [16]. However, trabecular bone adjusts to changes in loading during life with high sensitivity, which becomes visible in both the orientation and the dimensions of the trabeculae [12, 17]. Trabecular bone parameters furthermore scale allometrically [18–20]. In humans and other primates, the trabecular architecture in long bones is related to locomotor patterns [13, 21, 22]. The trabecular bone structure has been used as an indicator for reconstruction of locomotor habits in extinct taxa [23, 24]. However, it remains controversial whether trabecular bone reflects lifestyle in primates, and if so, how. In contrast to the above mentioned findings, other studies in anthropoids reported the femoral trabecular microstructure to be independent of locomotor behavior (e.g. [25]). A broader sampling of vertebrate bone morphology is required to address the question of how trabecular architecture is related to locomotor behavior.

Since research on lifestyle-related functional adaptation of bone microstructure mainly focuses on primates, little has been done in non-primate mammals so far. Recent studies in Xenarthra [26] and Odontoceti [27] expanded the sampling of mammalian 3D trabecular architecture in context of lifestyle. In rodents, however, the functional role of trabeculae is mostly studied experimentally. In rats and mice, the effect of physical exercise on trabecular bone has been investigated (e.g. [8, 9]). Experiments in rats have shown that unloading of hind limbs causes bone loss and degradation towards a lighter trabecular structure within three weeks [28]. Trabecular bone loss has also been shown to occur in hibernating ground squirrels [29]. Yet research on trabecular bone in rodents mainly focuses on the hormonal influence on bone microstructure, rather than on the biomechanical constraints [30–33].

This study investigates the effect of body mass and locomotor habits on femoral head trabecular bone structure in Sciuromorpha. Our aim is to elucidate the relationship of trabecular architecture to body mass and lifestyle-related loading conditions. In particular, we seek to identify whether the different loading regimes are reflected by trabecular architecture, testing the hypothesis that the more multidirectional loading in arboreal locomotion affects trabecular structure differently than the more unidirectional loading in fossorial lifestyle (cf. [6]). We furthermore aim at determining whether semifossorial species resemble rather the arboreal or fossorial sciuromorphs. Three-dimensional data analysis of high resolution computed tomography (CT-) scans was conducted in order to characterize trabecular bone in the femoral head of 69 sciuromorph species from different lifestyle categories. The body mass of these species ranged from approximately 16.5 g to 3000 g (*Myosciurus pumilio* [34] and *Marmota marmota* [35], respectively). A cubic volume of interest was selected in each femoral head for calculation of degree of anisotropy *DA*, main direction of trabeculae *MDT* (cf. [26]), connectivity density *ConnD*, mean trabecular thickness *TbTh*, mean trabecular separation *TbSp*, bone volume fraction *BV/TV* and bone surface density *BS/BV*. These trabecular parameters were used for analyses of the allometric and functional signal. We show that trabecular architecture relates to body mass. The data reveal functional signals related to lifestyle in some of the trabecular parameters. This study provides new findings on the significance of external loading patterns on trabecular bone structure in Sciuromorpha that will be valuable for experimental research aimed at elucidating the significance of bone microstructure in functional contexts.

## Methods

### Specimens

Sciuromorph femora have been chosen from the following museum collections for high resolution CT-scans: AMNH, American Museum of Natural History, New York City, USA; CeNaK, Centrum für Naturkunde, Universität Hamburg, Germany; MNHN, Muséum national d’Histoire naturelle, Paris, France; PMJ, Phyletisches Museum Jena, Germany; SMF, Naturmuseum Senckenberg, Frankfurt am Main, Germany; SMNS, Staatliches Museum für Naturkunde, Stuttgart, Germany; SNMNH, Smithsonian National Museum of Natural History, Washington, D.C., USA; UMMZ, University of Michigan Museum of Zoology, Ann Arbor, USA; ZMB, Museum für Naturkunde Berlin, Germany; ZSM, Zoologische Staatssammlung München, Germany.

**Fig. 1:**
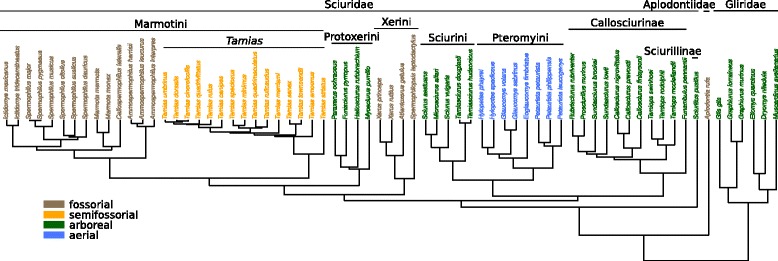
Phylogenetic tree of the sciuromorph species analyzed in this study. Colors indicate the lifestyle classification

See Additional file 1 for a detailed list of all specimens. CT-scans were acquired with resolutions of 6.4 - 42.5 *μ*m voxel size and saved as 8-bit or 16-bit grayscale image stacks in tif format. Juvenile specimens (defined as those with an epiphyseal plate at the femoral head visible on the scans) were excluded from the analyses. After applying quality thresholds (described below), the final dataset covered all three sciuromorph families (Sciuridae, Gliridae, Aplodontiidae), including 33 genera and 69 species. Lifestyle categorization based on previous literature [36–38] yielded the following composition (see Fig. 1): 27 arboreal species (representing Sciurillinae, Protoxerini, Sciurini, Ratufinae, Callosciurinae, Gliridae), 19 fossorial species (representing Xerini, *Aplodontia rufa*, Marmotini excl. *Tamias*), 15 semifossorial species (representing *Tamias*) and 8 aerial species (representing Pteromyini).

### 3D data analysis procedure

**Fig. 2:**
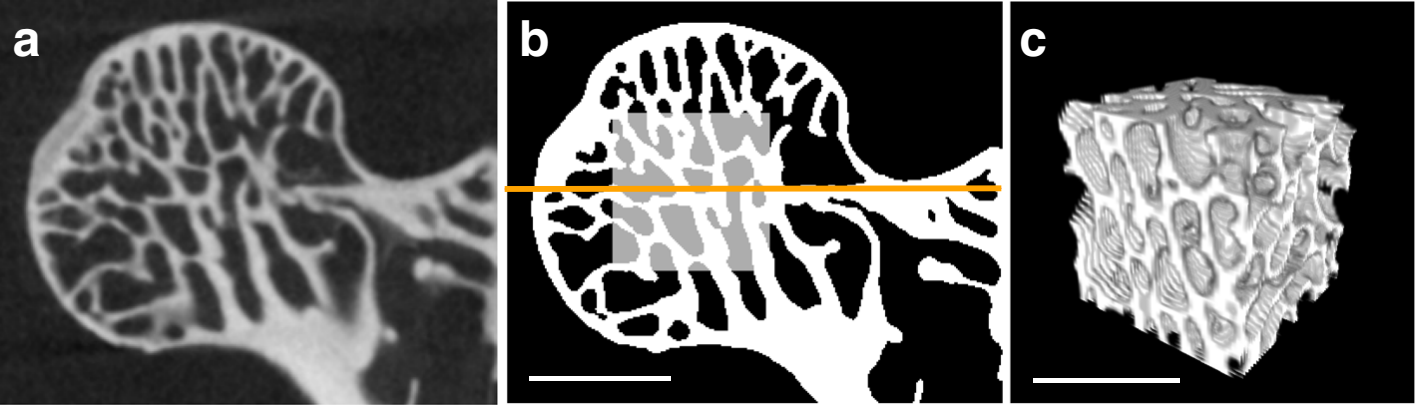
Workflow for the selection of the cubic volume of interest (VOI) in the femoral head. Selection of the VOI is shown with a specimen of *Tamias rufus* (SNMNH, 564127). **a** Micro-CT coronal slice, 16-bit grayscale image. **b** Selection of the cubic VOI in the center of the femoral head. The horizontal orange line indicates the central slice which divides the femoral head and the VOI into a proximal and a distal half. The VOI was expanded in three dimensions as much as possible without including cortical bone. **c** 3D view of the final selection after cropping the image stack. Analysis of trabecular architecture was executed on this substack. Note that these pictures serve as a demonstration for the VOI selection but do not conform with the proper analysis done on this specimen. Scale bars: 1 mm

Analysis of CT-scan data was done using the Fiji contribution of ImageJ vers. 1.51k [39, 40] and the plugin BoneJ vers. 1.4.2 [41]. An analysis Java script (Additional file 2) was composed and applied to all specimens. Prior to the analysis, each femur was oriented within the stack coordinate system using the ’Rotate’ function and the ’Reslice’ function in Fiji. The proximodistal axis of the femoral shaft was thereby aligned along the z-axis of the coordinate system (defined then as the proximodistal axis also for the femoral head). The mediolateral and anteroposterior axes were aligned along the x- and y-axis, respectively, using the femoral condyles as reference. A cubic volume of interest (VOI) was then selected in the center of the femoral head (Fig. 2). The central slice was defined as the plane that divides the femoral head into a distal and a proximal half (Fig. 2b). The VOI originated from this slice and was expanded as a cube in all three dimensions as much as possible without including cortical bone. The center of the VOI on the mid-slice was thereby positioned such that it allows for a maximum spread over the eight corners of the VOI. Scans with an insufficient resolution were excluded. We used a relative resolution as the number of pixels covering the mean trabecular thickness *TbTh* (relative resolution = *TbTh* /pixel size) [42, 43] and discarded all scans which fell below a relative resolution of five (cf. [42]). To allow for application of phylogenetically informed methods, we included only those species in the analysis for which the exact position within the phylogenetic tree is known (shown in Fig. 1).

All further analyses were done using the BoneJ plugin. For preparation the VOI was binarized (using the ’Optimise Threshold’ function) and purified (’Purify’ function). The calculated parameters for characterization of the trabecular architecture were the following: degree of anisotropy *DA* with vector of main direction of trabeculae *MDT* (’Anisotropy’ function), connectivity density *ConnD* (’Connectivity’ function), mean trabecular thickness *TbTh* and trabecular separation *TbSp* (’Thickness’ function) as well as bone volume fraction *BV/TV* and bone surface density *BS/BV* (’Volume Fraction’ function and ’Isosurface’ function, respectively). *DA* is calculated in BoneJ by using the mean intercept length method [44]. The parameter indicates to what extent the trabeculae are oriented in a preferential direction. The measure as used here ranges from zero (fully isotropic, no preferred direction) to one (fully anisotropic). The main orientation of trabeculae *MDT* is given by the eigenvector corresponding to the lowest eigenvalue, which reflects the major axis of the ellipsoid defining the trabecular anisotropy [44]. All vectors were projected onto the proximal hemisphere of the femoral head and those of right femora were mirrored to the left side. *MDT* is a three-dimensional parameter (*x*,*y*,*z*) with *x* describing the mediolateral, *y* the anteroposterior and *z* the proximodistal contribution to the main orientation. Note that *MDT* can be compared among the specimens because the femora are all oriented in the same reference coordinate system given by the image axes. *ConnD* is calculated in BoneJ using the Euler characteristic *Δχ* with *ConnD*=(1−*Δχ*)/stack volume. The parameter represents a measure for the number of trabeculae per volume [45]. Accurate analysis of trabeculae implies a continuum assumption for the trabecular bone, which requires a minimal number of trabeculae within the VOI [46]. Thus all samples with an absolute connectivity (*Conn*) of less than 50 were discarded. *TbTh* and *TbSp* are calculated as the mean thickness of the fore- and background in the 3D VOI stack. Thickness at a point is thereby defined as the diameter of the greatest sphere fitting within fore- or background structure and containing this point. *BV/TV* is measured using a voxel-based algorithm that calculates the ratio of foreground voxels (representing bone tissue) and total number of voxels in the sample. The measure gives the proportion of volume that is occupied by bone. *BS/BV* was calculated by measuring the bone surface area and dividing it by the bone volume of the sample.

### Phylogenetic tree

The sciuromorph phylogenetic tree used herein (Fig. 1) is based on a previous study [47] and complemented with species from the TimeTree database [48] (Gliridae, *Tamiops swinhoei*, *Tamiops rodolphii*, *Aplodontia rufa*, *Heliosciurus rufobrachium*) using Mesquite version 3.04 [49]. Given the apparent correlation of the lifestyle categories with the phylogeny (see Fig. 1), we conducted subsequent analyses with phylogenetically informed methods.

### Allometry analysis

For all trabecular parameters the relation with VOI edge length *vl* was tested. Since the actual individual body masses were unknown for the specimens, *vl* served as a proxy for body mass. A test with available body masses taken from literature for a subset of 64 species (93% of the species covered in this study) revealed significant correlation of *vl* with body mass (*r*^2^=0.92, *p*<10^−44^ with *r* being the Pearson correlation coefficient). Most importantly, *vl* scales isometrically with body mass *m*. We found *m*∝*vl*^2.95^ with *m*∝*vl*^3^ being the expected scaling under isometry. The observed scaling (exponent 2.95) did not differ significantly from the isometric (exponent 3) case (*p*=0.56). Thus, we consider *vl* a suitable proxy for body mass, a predominant factor in loading.

The relation of any trabecular parameter *tp* with *vl* was quantified by calculating the scaling exponent *a* such that *tp*∝*vl*^*a*^ and accordingly *log*(*tp*)∝*a*·*log*(*vl*). All raw data were natural log-transformed beforehand. Linear regression analysis was performed in R vers. 3.4.2 [50] using the *gls()*-function in the *’nlme’* package vers. 3.1.131 [51]. Regressions were done phylogenetically informed (*corPagel()*-function in the *’ape’* package vers. 5.0 [52]). The linear correlation between the log-transformed data and log-transformed *vl* was quantified by calculating the slope *a* and the coefficient of determination *r*^2^. To identify the type of scaling (negative or positive allometry or isometry), we tested for significant deviation of the observed scaling exponent (*a*_*obs*_) from the expected scaling exponent for the case of isometry (*a*_*iso*_). To do so, we set the isometric regression line as baseline (such that the isometric slope becomes zero for all parameters) by subtracting the value expected under isometry (*vl*·*a*_*iso*_) from each data point. We then performed the regression as described above, testing the null hypothesis *a*_*obs*_=0. Slopes significantly higher or lower than zero indicate positive or negative allometry, respectively. Slopes not significantly deviating from zero indicate isometry or near-isometry. For *BV/TV* and *DA*, *a*_*iso*_=0 (Table 1), as these measures are dimensionless ratios, which do not change with increasing size under assumption of isometry. On the other hand *TbTh* and *TbSp* (unit: *length*^1^) are absolute linear measures which would increase under isometry with *a*_*iso*_=1. For *BS/BV* (unit: *length*^−1^) applies *a*_*iso*_=−1 (the volume *BV* increases more rapidly than *BS*). The isometric scaling exponent for *ConnD* (unit: *length*^−3^) is *a*_*iso*_=−3. Under isometric growing of the trabeculae (model of scaling with constant trabecular geometry [53]), fewer trabeculae fit into one unit volume with increasing size. For each parameter, a *p*-value was calculated for testing the null hypothesis *a*_*obs*_=*a*_*iso*_ as described above. Thus, isometry was assumed when *p*>0.05. Otherwise we assumed positive allometry for *a*_*obs*_>*a*_*iso*_ and negative allometry for *a*_*obs*_<*a*_*iso*_.

**Table 1:** Allometry in the sciuromorph femoral head trabeculae - relation between trabecular parameters and VOI edge length

Parameter	*a*_*iso*_	*a*_*obs*_	*p*-value	Scaling
*BV/TV*	0	0.166	0.004	+allo
*ConnD*	−3	−2.138	1.2×10^−5^	+allo
*BS/BV*	−1	−0.829	0.013	+allo
*DA*	0	−0.193	1.1×10^−5^	−allo
*TbSp*	1	0.593	7.4×10^−8^	−allo
*TbTh*	1	0.835	0.033	−allo

*a*_*iso*_ - expected scaling exponent under isometry

*a*_*obs*_ - observed scaling exponent

*p*-value - tests null hypothesis *a*_*obs*_=*a*_*iso*_

+/ −allo - positive or negative allometry

### Lifestyle analysis

In order to test for differences in trabecular architecture among the four lifestyle categories (arboreal, fossorial, aerial and semifossorial), we performed a pairwise statistical analysis on the individual trabecular parameters.

The significance level for all statistical tests was fixed at *α*=0.05. Each pair of lifestyles was compared for each *tp* by regression in R vers. 3.4.2 [50] with the *gls()*-function in the *’nlme’* package vers. 3.1.131 [51] while taking correlation with phylogeny into account (*corPagel()*-function in the *’ape’* package vers. 5.0 [52]). Since all parameters were correlated with *vl*, the body mass proxy was included as covariate in the function to separate the influence of lifestyle and *vl* on the parameter (R formula: *tp*∼*vl*+*lifestyle*). Because this procedure of multiple statistical tests increases the rate of type I errors, we corrected the *p*-values with a Benjamini-Hochberg method [54] (*p.adjust()*-function in the *’stats’* package in R). Only the corrected *p*-values are reported herein. Since this analysis procedure takes correlation with phylogeny and body mass into account, it is ensured that the confounding effect of these factors is reduced. Hence, we are able to analyze and interpret the data with regard to an effect of lifestyle on trabecular properties.

## Results

### Main orientations of trabeculae are distributed around anteroposterior axis

**Fig. 3:**
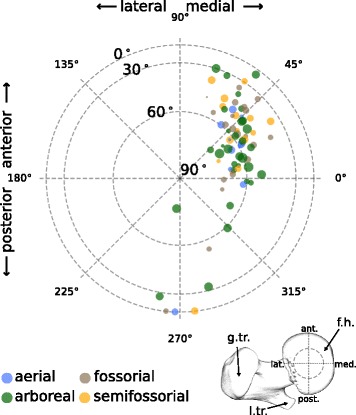
Trabecular orientation. The main direction of trabecular anisotropy (*MDT*) is represented by vectors originating in the center of the femoral head and projected onto its proximal hemisphere. A femur, seen in proximal view, is depicted at lower right to illustrate the orientation (g.tr./l.tr., greater/lesser trochanter; f.h., femoral head). Data points close to the center are oriented more proximodistally. Colors indicate the lifestyle categories, circle size the degree of anisotropy (*DA*), ranging from 0.451 (smallest circle) to 0.883 (largest circle). The data show the results for left femora (right femora were mirrored)

In order to investigate how the trabeculae are oriented within the femoral head, their main direction *MDT* was displayed as vectors in spherical coordinates (Fig. 3). These vectors, originating in the center of the VOI, were projected onto the proximal hemisphere of the femoral head. We observed no apparent differences in *MDT* among the lifestyle categories (Fig. 3). The trabeculae of most specimens were oriented in the proximo-antero-medial direction. The main variation in the whole dataset was observed along the anteroposterior axis.

### Femoral head trabecular bone parameters scale allometrically

To test whether the trabecular parameters depend on body mass, the correlation of each of them with the edge length *vl* of the cubic VOI was analyzed by calculating the coefficient of determination *r*^2^ and the scaling exponent *a*. To identify allometric or isometric scaling, we compared the observed scaling exponents with the expected scaling exponents for the case of isometry (*a*_*iso*_, see Table 1).

**Fig. 4:**
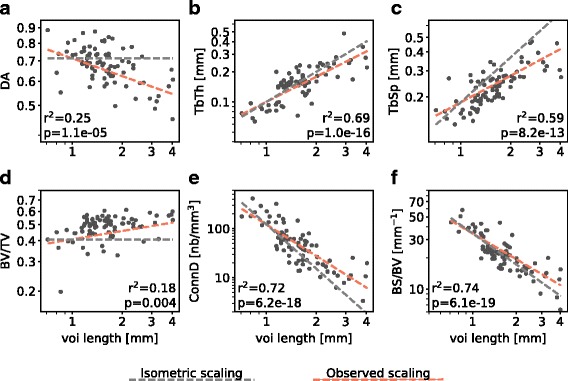
Allometric scaling in trabecular parameters. Each plot shows the relation between the trabecular parameter and edge length of the cubic VOI (*vl*), the latter serving as proxy for body mass (see text). Raw data were plotted on log-log scale. *r*^2^ - coefficient of determination, *p* - *p*-value of the regression. **a-c** Degree of anisotropy *DA*, trabecular thickness *TbTh* and trabecular separation *TbSp* scale with negative allometry. **d-f** Bone volume fraction *BV/TV*, connectivity density *ConnD* and bone surface density *BS/BV* scale with positive allometry

We observed positive allometry for *BV/TV*, *ConnD* and *BS/BV* and negative allometry for *DA*, *TbTh* and *TbSp* (Table 1, Fig. 4). We conclude from the negative allometry in absolute *TbTh* and *TbSp* that relative *TbTh* and *TbSp* were lower with higher *vl*. Thus, trabeculae tend to be relatively thinner and more densely packed in larger animals (higher *ConnD*). Comparison of the scaling of *TbSp* and *TbTh* revealed that *TbTh* increased more rapidly with *vl* than *TbSp* did, which is consistent with the positive allometry in *BV/TV*. Taken together, these results reveal a significant allometric signal in all six trabecular parameters.

### Femoral head trabecular bone reflects lifestyle

**Fig. 5:**
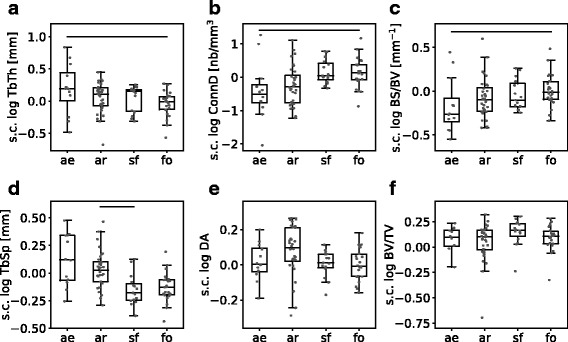
Lifestyle-related adaptations of trabecular parameters. The boxplots illustrate distributions of the size-corrected (s.c., residuals after allometry regression) data of the trabecular parameters in the lifestyle groups aerial *ae*, arboreal *ar*, semifossorial *sf* and fossorial *fo*. Significant differences between groups, as revealed by phylogenetically informed regression, are indicated by the horizontal lines (*p*-values were corrected for multiple testing, for detailed explanation see text). **a-c** Aerial species are distinguished from fossorial ones by a higher trabecular thickness *TbTh*, lower connectivity density *ConnD* and lower bone surface density *BS/BV*. **d** Trabecular separation *TbSp* is higher in arboreal species than in semifossorial ones. **e-f** Degree of anisotropy *DA* and bone volume fraction *BV/TV* do not show significant functional signals

To analyze how the individual trabecular parameters relate to lifestyle, a phylogenetically informed pairwise statistical analysis of the log-transformed data was performed by comparing each two of the four lifestyle groups while accounting for the effect of mass. We identified functional signals in *TbTh*, *ConnD*, *BS/BV*, and *TbSp* (Fig. 5a-d): Aerial species are distinguished from fossorial ones by a higher *TbTh* (*p*_(*ae*,*fo*)_=2×10^−7^), a lower *ConnD* (*p*_(*ae*,*fo*)_=0.0007) and a lower *BS/BV* (*p*_(*ae*,*fo*)_=2.2×10^−5^). Arboreal species have a higher *TbSp* than semifossorial species have (*p*_(*ar*,*sf*)_=4.1×10^−5^). *DA* and *BV/TV* showed no significant differences among lifestyles (Fig. 5e-f). However, although not being significant, *DA* tends to be higher in arboreal species. When considering a ’lifestyle gradient’ as the sequence aerial > arboreal > semifossorial > fossorial, we observe the general trend of decreasing *TbTh* and *TbSp* and increasing *ConnD* and *BS/BV* towards the fossorial lifestyle (Fig. 5). When comparing semifossorial species with arboreal and fossorial ones, we observed that they generally resemble the fossorial sciuromorphs more closely than they do the arboreal ones (mostly apparent in *ConnD*, *TbSp* and *DA*, see Fig. 5). Taken together, these results reveal lifestyle-related differences in four of six analyzed trabecular parameters (*TbTh*, *ConnD*, *BS/BV*, and *TbSp*).

## Discussion

The aim of this study was to characterize trabecular microstructure of the sciuromorph femoral head and to investigate its relation to body mass and locomotor behavior. The sciuromorph trabeculae are preferentially oriented in proximo-antero-medial direction within the femoral head and are distributed around its anteroposterior axis. The tested trabecular parameters show different scaling effects: whereas *DA*, *TbTh* and *TbSp* scale with negative allometry, a positive allometry was revealed for *BV/TV*, *ConnD* and *BS/BV*. Phylogenetically informed analysis identified functional signals in *TbTh*, *TbSp*, *ConnD* and *BS/BV*. It distinguished aerial Sciuromorpha from fossorial ones (higher *TbTh*, lower *ConnD* and lower *BS/BV* in aerial species) and arboreal Sciuromorpha from semifossorial ones (higher *TbSp* in arboreal species). Semifossorial sciuromorphs resembled the fossorial species rather than the arboreal ones.

The observed proximomedial orientation of the trabeculae is on average roughly parallel to the femoral neck. The anteroposterior distribution of main trabecular orientation is in agreement with observations in primate femora [10, 22]. We suggest that this pattern of trabecular orientation may indicate that the predominant pro-/retraction angle at high loading phases during locomotion is a determining factor of this trabecular orientation, both in primates and sciuromorphs.

Allometric scaling has been observed in trabecular architecture of different bones in a large variety of taxa [18–20]. The allometric scaling patterns of trabecular parameters observed for the sciuromorph femoral head trabeculae conform with observations in primates [19]. Other studies reported *BV/TV* and *DA* to be independent of body size [18, 20]. Our result for the scaling of *ConnD* (*a*=−2.138) qualitatively conforms with the observation reported for the primate femur (*a*=−1.22, see [19]). However, in that previous study, *ConnD* has been considered as a shape variable which does not change with body size and scales with *a*=0 in the isometric case. Hence, the scaling has been reported as negative allometry. This would be the case if trabecular size (e.g. *TbTh*) remains constant with increasing body size and solely the trabecular number increases (model of ’constant trabecular size’ as against ’constant trabecular geometry’ [53]). If *ConnD* is interpreted as number of trabeculae per volume, as herein, and trabecular size scales isometrically, the isometric scaling exponent should be *a*=−3 and the scaling be positively allometric. Positive allometry in *BV/TV*, as observed herein, is also observed in other rodents, mainly due to variation in trabecular number rather than variation in *TbTh*, the latter being the main reason in humans [20].

Previous studies drew different conclusions as to whether trabecular bone structure reflects locomotor habits. The femoral neck trabecular architecture in nonhuman anthropoid primates has been reported by one study to be independent of locomotor mode, phylogenetic background and even body mass [25]. In contrast, other studies, including the present one, observed a relation between trabecular bone and locomotion [10, 13, 26, 55, 56]. We identified *TbTh*, *TbSp*, *ConnD*, and *BS/BV* in the sciuromorph femoral head microstructure to be correlated with locomotor habits. It has also been shown that the proximal femur of human foraging populations has higher *BV/TV* and *TbTh* and a lower *BS/BV* compared to agriculturalist groups, revealing a correlation between high mobility and relative bone stiffness [13]. Other comparative studies observed functional adaptations related to leaping behavior in *BV/TV*, *DA* or *MDT* in different primate species [10, 55, 56]. Furthermore, it has been shown that trabecular bone reflects basic primate locomotor categories, such as brachiation, quadrupedal/bipedal walking, climbing, and jumping [21, 22]. Trabecular bone has already been used in reconstructing locomotor modes of fossil primates [23, 56].

However, the approach of using bone microstructure as an indicator for locomotor habits has limitations. In addition to body mass and lifestyle, ontogenetic stage of an animal might also influence trabecular architecture (e.g., [57]). In collection-based research age cannot be considered, as the precise age of death of the specimens is usually not known. However, it has been shown in mice that trabecular parameters change mostly during the first six months of life and then pass into a stable phase [58]. By excluding specimens whose epiphyseal plate was not fused, we avoided including too young specimens that might not have reached that state. For studying allometry, an estimation of body mass is desirable. Equations for that purpose, based upon bone articular and diaphyseal structure, exist but should be used cautiously [59]. Thus, directly available measures, such as femoral head dimensions, are commonly used as body size proxy (e.g., [18, 19]). In this study, we decided to use the length of the VOI (*vl*) as a proxy for body size, as we could show that this parameter scales isometrically with body mass, a predominant factor in loading. The architecture of trabecular bone is highly variable not only among species but even among individuals of the same species [24, 53]. Our dataset, composed of solely one or two individuals per species, does not allow an assessment of this intraspecies variance. However, since the dataset comprises a broad sampling on the level of genera, lifestyle and body mass, it still allows us to draw conclusions about trabecular structure in a general functional context.

This study conducted research on lifestyle-related functional adaptation of trabecular architecture in Sciuromorpha and thereby supplements the few non-primate studies of that kind. The different body sizes and locomotor habits within the Sciuromorpha account for naturally varying characteristic loading patterns acting on the bones of the animals. These loading patterns conferred structural differences in the femoral head trabecular microstructure, as revealed by phylogenetically informed GLS regression.

The observed trends support our hypothesis that the loading patterns related to climbing and digging behavior are differently reflected by trabecular parameters. The apparent bone density is related to bone stability [60] and biomechanical loading [13]. This may be due to the fact that trabecular bone, a material which needs to absorb energy during impact loading, can increase stability and absorbing capacity through increasing the apparent bone density [61]. This can be achieved through (1) an increase in trabecular number, as typical for rodents, or (2) an increase in trabecular thickness, as typical for humans [13, 20]. When considering a ’lifestyle gradient’ (without relation to the evolutionary history) as the sequence aerial > arboreal > semifossorial > fossorial, we observed the mechanism (1) (increase in trabecular number, thus in *ConnD*) as a trend toward fossorial sciuromorphs and mechanism (2) (increase in *TbTh*) as a trend towards aerial sciuromorphs. Which functional constraints constitute these different mechanisms of structural adaptation? On the one hand, in aerial sciuromorphs, bones (while needing to retain light-weight structure) need to resist high peak loads when the animals land after gliding [7]. Thus, the bone is loaded with a low frequency, but with a high magnitude, a loading pattern that particularly triggers adaptive response in bones [62, 63]. Here, the need to avoid fractures within the trabecular network may dominate and account for the mechanism of increased trabecular thickness in aerial sciuromorphs (cf. [22]). On the other hand, fossorial species are expected to experience lower peak forces, but at a higher frequency. The hindlimbs provide propulsion during running and ensure stability during digging activity of the forelimbs [64]. Here, the need to retain more sustained material stability against moderate loading may account for the mechanism of increased trabecular number (cf. [11, 21]). Thus, trabeculae in fossorial sciuromorphs are thinner and more closely packed (as observed as a trend in semifossorial species as well), a pattern that we found also for large-sized sciuromorphs when analyzing allometric scaling of trabeculae. This comes along with an increased *BS/BV*, which may facilitate transfer of minerals and calcium mobilization at the surfaces of trabeculae [53] and might thus be an adaptive mechanism observed both in large sized animals and in animals with particularly sustained physical activity.

## Conclusions

This study characterizes trabecular architecture and its relation to body mass and locomotor behavior in the sciuromorph femoral head. The results reveal allometric scaling in the set of trabecular parameters analyzed herein. The trabecular microstructure reflects lifestyle-related functional signals. Similar results have been documented in a few mammalian clades (primates [13, 21, 22], Odontoceti [27], Xenarthra [26]) and thus suggest to provide important insight into the functional morphology of mammals in general.

Subsequent research ought to focus on identifying the different mechanisms of functional adaptation in trabecular microstructure in animals of different body sizes and lifestyles. Our findings could be verified with experimental studies including setups that imitate different habitats and therefore different loading patterns applied on living Sciuromorpha. Thus, one could investigate experimentally whether trabecular parameters are indeed differently affected by loading patterns related to climbing and digging, as suggested in this study. This would further elucidate the functional significance of bone internal structure in Sciuromorpha.

## Additional files

Additional file 1 List of all analyzed specimens and their trabecular parameters. Complete list of the specimens analyzed in this study (.csv-file), including information about their origin (museum and collection number), their relative resolution, the CT-scanner, their sex, their assigned lifestyle category and the raw data characterizing the VOIs (*MDT*, *DA*, *ConnD*, *TbTh*, *TbSp*, *BV/TV*, *BS/BV*). (CSV 13 kb)

Additional file 2 3D data analysis Java script for BoneJ in Fiji. Java script, implemented for the extraction of trabecular parameters of an image stack with the BoneJ plugin in Fiji. With BoneJ being installed it is ready to use after opening it in Fiji (File/Open...). (0.763 kb)

## Abbreviations

BS/BV: Bone surface density
BV/TV: Bone volume fraction
ConnD: Connectivity density
CT: computed tomography
DA: Degree of anisotropy
MDT: Main direction of trabeculae
TbSp: Trabecular separation
TbTh: Trabecular thickness
tp: Trabecular parameter
vl: VOI edge length
VOI: Volume of interest
